# Interactions of amino acids with aluminum octacarboxyphthalocyanine hydroxide. Experimental and DFT studies

**DOI:** 10.1007/s00894-017-3222-2

**Published:** 2017-02-04

**Authors:** Marta Kliber-Jasik, Małgorzata A. Broda, Anna Maroń, Joanna Nackiewicz

**Affiliations:** 1 Faculty of Chemistry, Department of Physical Chemistry and Molecular Modeling, University of Opole, Oleska 48, Opole, 45-052 Poland; 2 Institute of Chemistry, Department of Crystallography, University of Silesia, 9th Szkolna St., Katowice, 40-006 Poland

**Keywords:** Aluminum octacarboxyphthalocyanine hydroxide, Amino acid, Protein, Photodynamic therapy, DFT calculations, TD-DFT spectra

## Abstract

The influence of albumin and amino acids (l-serine, glycine, l-histidine, l-tryptophan, l-cysteine) on the properties of aluminum octacarboxyphthalocyanine hydroxide (Al(OH)PcOC) was investigated in a phosphate buffer (pH 8.0). Particular attention was paid to the spectroscopic properties and photostability of Al(OH)PcOC. The effect of albumin or amino acids on the photodegradation of Al(OH)PcOC was examined in water using red light: 685 nm and daylight irradiation. Analysis of kinetic curves indicated that interaction with those molecules increases the photostability of Al(OH)PcOC. The molecular structure of Al(OH)PcOC complexes (in vacuum and in water) with axially or equatorially coordinated amino acids was studied by the B3LYP/6-31G* method, and the effects on molecular structure and electronic absorption spectrum were investigated on the basis of the density functional theory. The calculation results revealed that axial coordination significantly reduces the non-planarity of the phthalocyanine ring, and, thus, alters the electronic structure. On the other hand, hydrogen bonding of phthalocyanine side COOH groups with amino acids, in equatorial complexes, does not change the structure within the center of the phthalocyanine, and causes only a slight increase in UV–vis bands intensity, which is in perfect agreement with experimental data.

**Electronic supplementary material:**

The online version of this article (doi:10.1007/s00894-017-3222-2) contains supplementary material, which is available to authorized users.

**Graphical abstract:**
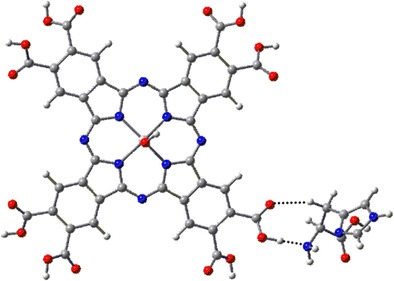
Structure of equatorial complex of Al(OH)PcOC with l-histidine calculated byB3LYP/6-31G(d) method.* Dotted lines* H-bonds

## Introduction

Phthalocyanines (Pcs) are structural analogues of porphyrins, but, in contrast to the latter, they are not found in nature and are obtained solely by chemical synthesis [1]. Pcs compounds can form complexes with most elements [2]. Due to their spectroscopic and photoelectric properties, as well as excellent chemical and thermal stability in different environments, substituted Pcs have been studied extensively, especially in recent years [3]. Metallophthalocyanines (MPcs) have been used, for example, as oxidation catalysts, chemical sensors, organic light-emitting diodes, semiconductors, molecular organic photovoltaics, photosensitizers in dye solar cells, photoactive elements in photocopiers, electrochromic displays and in liquid crystalline materials [2, 4]. In recent years, Pcs have proved promising as second generation photosensitizers for photodynamic therapy (PDT) owing to their strong absorption in the phototherapeutic window 600–900 nm, ease of modification, high efficiency of generating reactive oxygen species (ROS), low dark toxicity and high phototoxicity [5–9]. The complexes of Pcs containing metals such as Al^3+^, Ga^3+^ and Zn^2+^ are particularly interesting because these molecules have a high triplet quantum yield and long triplet lifetimes. These properties are necessary for high singlet oxygen quantum yields, and guarantee high cytotoxicity against tumors [10, 11]. Unsubstituted Pcs have two major limitations, i.e., low water solubility and a strong tendency to aggregate, which causes problems for their application in medicine [12, 13]. Nevertheless, the solubility of Pcs can be improved by the introduction of suitable functional groups to increase their hydrophilicity and make solvation easier. Increased solubility and reduced aggregation are very important requirements in PDT applications because aggregates shorten the triplet-state lifetime and reduce the singlet oxygen quantum yield [14]. From the point of view of medical applications, is it important to synthesize water-soluble Pcs so that drugs can be injected directly into the patient’s bloodstream [15–18].

The photostability of MPcs is of fundamental importance for their use as photosensitizers in PDT. The term photodegradation (photobleaching) is generally used and defined in the literature as a decrease in the absorbance (of both the Q and B bands) caused by exposure to light. The phenomenon of photobleaching is dependent on many factors, e.g., the nature of substituents, dye concentration, the tendency to aggregate, and the type of solvent used. [19–21].

**Fig. 1:**
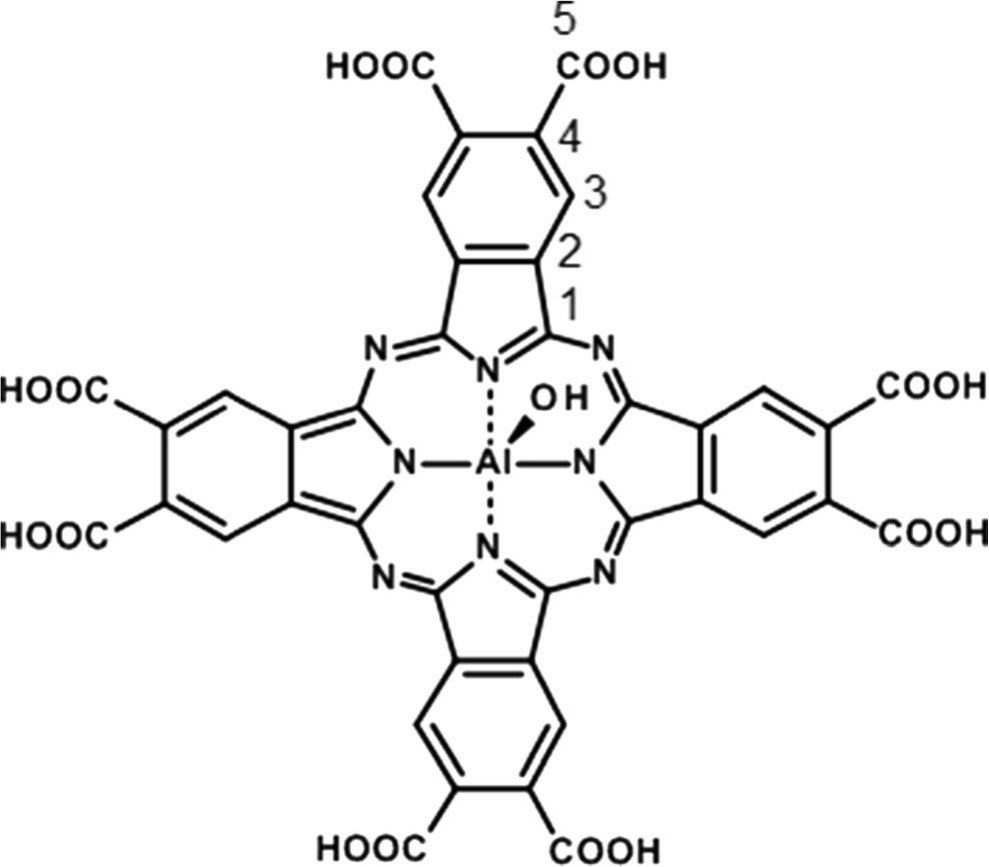
Structure of aluminum octacarboxyphthalocyanine hydroxide [Al(OH)PcOC]

Aluminum octacarboxyphthalocyanine hydroxide [Al(OH)PcOC] (Fig. 1) has been regarded as a potential agent for photodynamic therapy [22, 23]. However, a proper candidate for PDT should be photochemically active, stable, and occur in a monomeric form. Obviously, a detailed knowledge about the interaction of compounds used for in vivo administration with living cell components, including amino acids and proteins, is required.

The objective of this paper was to investigate the interactions between Al(OH)PcOC and amino acids (glycine, l-serine, l-cysteine, l-tryptophan, l-histidine) or albumin. We investigated especially the tendency for aggregation and the photochemical stability of Al(OH)PcOC in the presence of albumin or selected amino acids in phosphate buffer. Moreover, density functional theory (DFT) calculations were performed in order to decide how amino acids interact with the studied Al(OH)PcOC. The optimized structures of different types of Al(OH)PcOC complexes with selected amino acids in environments of diverse polarity were obtained at the B3LYP/6-31G* level of theory. We analyzed the impact of hydrogen bonds and electrostatic interactions on complex stability. We also demonstrated the influence of amino acids on the TD-DFT predicted UV–vis spectra of Al(OH)PcOC.

## Experimental

Details of the experimental and theoretical studies are included in the Supplementary materials.

### Synthesis of Al(OH)PcOC

Al(OH)PcOC was synthesized, purified and characterized according to published methods [24, 25]. In a first step, a mixture of pyromellitic dianhydride (benzene-1,2,4,5-tetracarboxylic dianhydride) (11.5 mmol), urea (0.22 mol), AlCl_3_ (23.5 mmol) and dibutylurea (DBU; 0.1 g) were placed in a 100 ml two-necked round bottom flask fitted with a reflux condenser and a thermometer. The mixture was heated to 250 °C until the reaction mixture fused. The fused product was washed with water, acetone and HCl (3 M). In a second step, hydroxyaluminum octa-4,5-carboxyphthalocyanine tetraimide was hydrolyzed by boiling with 10 ml 20% H_2_SO_4_ (72 h). The resulting suspension was filtered, and the precipitate was washed with 100 ml portions of hot 5% H_2_SO_4_ and water. The product was purified by repeated chromatography on Al_2_O_3_ using a NaOH solution (1%) as the eluent.

Yield: (8%). Calc. for C_40_H_17_N_8_O_17_Al (9H_2_O): C, 44.87; H, 3.29; N, 10.47. Found: C, 44.83; H, 3.22; N, 10.71. IR (KBr, cm^−1^): 3413, 1707, 1580, 1454, 1315, 1264, 1226, 1137, 1076, 733, 629. UV–vis (DMSO), λ_max_ (nm) (log Ɛ): 361 (4.74), 629 (4.44), 701 (5.16). Double charged ions in time-of-flight–electrospray ionization mass spectorometry (TOF-ESI MS) spectrum corresponded to Al(OH)PcOC anions formed due to the dissociation of two protons, *m/z*: 453.0; 453.5; 454.0; 454.5; 455.0 [M − 2H]^2−^. ^1^H NMR (400 MHz, D_2_O, ppm): 9,53 (8H, s, Pc-H), 4.68 (8H, s, carboxylic-H), ^13^C NMR (400 MHz, D_2_O, ppm): 150.92 (C-1), 121.52 (C-2), 140.00 (C-3), 136.46 (C-4), 176.85 (C-5).

## Results and discussion

### Aggregation studies

The properties of Pcs depend largely on aggregation. Pcs, due to strong π–π interaction and the flatness of the aromatic cores, have a tendency to aggregate in solutions, which, for this type of complex, is a well-known feature widely described in the literature [12, 26–29]. In PDT, association is a negative phenomenon, i.e., it has an unfavorable effect on the physicochemical features of photosensitizers and decreases efficiency of generating ROS, which causes a meaningful weakness in the cytotoxicity of a potential drug [30–32]. In solution, the formation of associated molecules can be an effect of limited solubility [26]. The carboxylic groups in the peripheral benzene rings of the Al(OH)PcOC molecules make the compound well soluble in water. The research indicated that only monomers could produce highly oxidative cytotoxic media, i.e., free radicals or singlet oxygen, efficiently [30, 33].

**Fig. 2:**
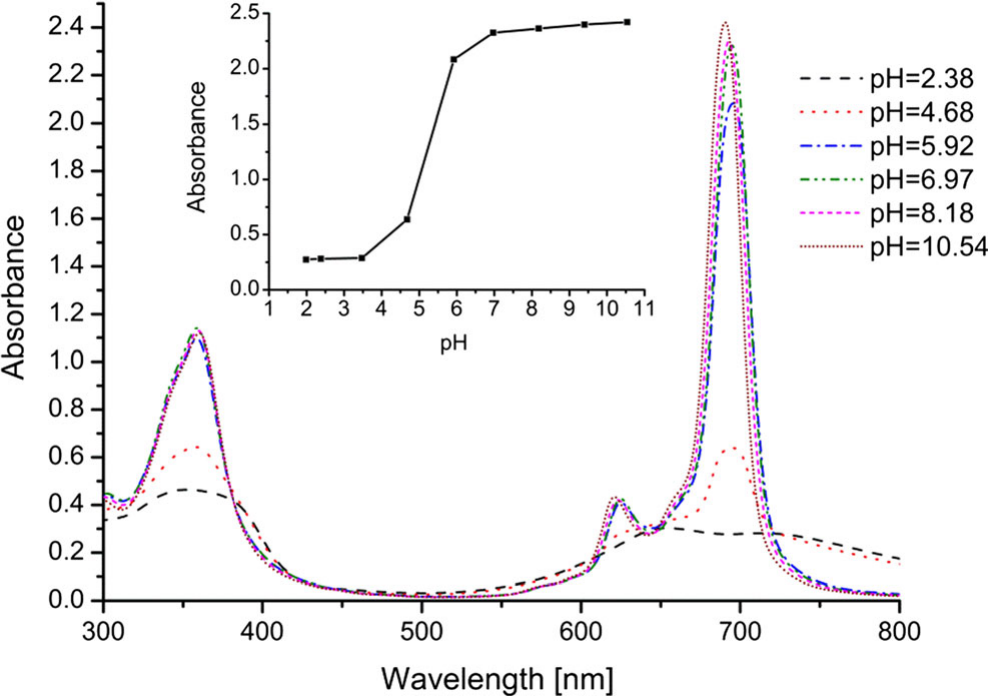
Influence of pH on the UV–vis spectrum of Al(OH)PcOC, concentration of Al(OH)PcOC was 1⋅10^−5^ mol/dm^3^, Britton-Robinson buffer solutions were used. In inset the influence of pH on absorbance of Al(OH)PcOC solutions in the Q-band region

The effect of pH on aggregation was tested for Al(OH)PcOC, which, like ZnPcOC [26], has a higher tendency to associate in acidified solutions than in water. It was found that absorbance of Al(OH)PcOC solutions depended on pH. Figure 2 shows that, in acid solutions (pH 2–5), the absorption spectrum is typical for phthalocyanine aggregates. In solutions of pH > 5, a considerably weaker band of vibronic character appeared, with λ_max_ = 625 nm, while at pH ≥ 7 Al(OH)PcOC occurs predominantly in the monomeric form due to the electrostatic repulsion of negatively charged carboxylic groups.

**Fig. 3:**
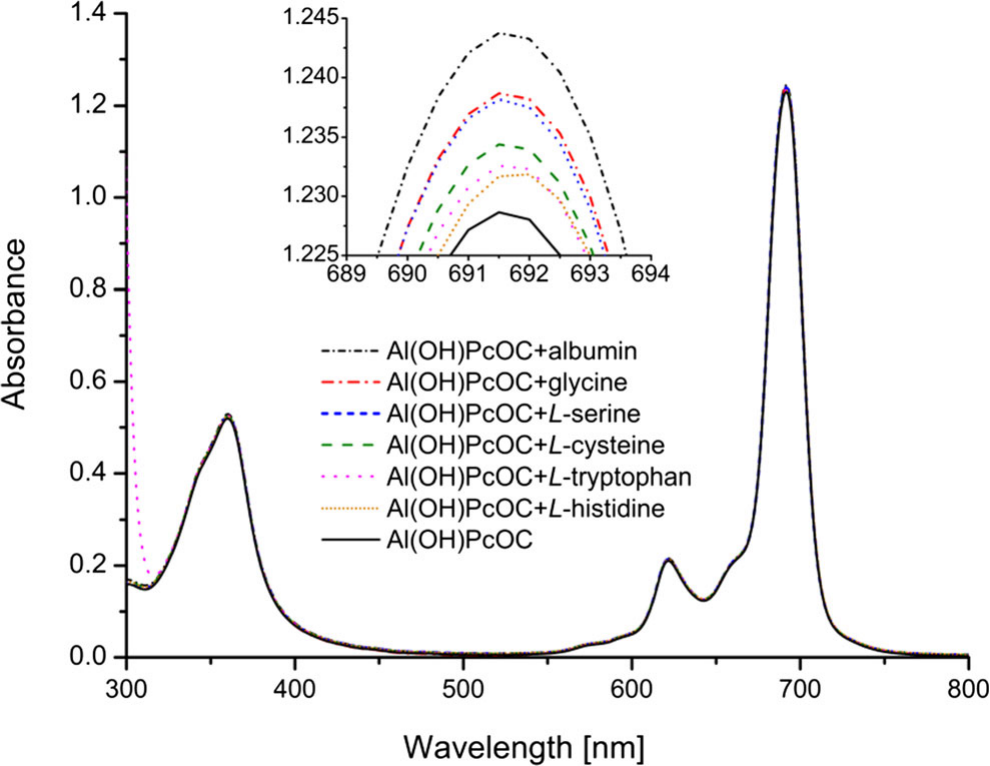
The influence of albumin or amino acids on the UV–vis spectrum of Al(OH)PcOC. The concentrations of Al(OH)PcOC, amino acids and albumin were 5 × 10^−6^ , 2 × 10^−3^ mol/dm^3^, and 0.27 mg/ml, respectively

Figure 3 demonstrates the influence of albumin and amino acids on the UV–vis absorption spectrum of Al(OH)PcOC at pH 8.0. The UV–Vis spectrum of Al(OH)PcOC exhibited absorption in the Q-band region (λ_max_ = 691.5 nm) characteristic of monomeric metallophthalocyanine, accompanied by a less intense vibronic band at λ_max_ = 621.5 nm and in the B band region at λ_max_ = 360 nm.

The presence of selected amino acids or albumin does not have an impact on the position of the bands in the Soret region (λ_max_ = 360 nm) or in the Q region (λ_max_ = 621.5 and 691.5 nm), just as in the case of zinc octacarboxyphthalocyanine [34]. Nevertheless, after adding albumin or amino acids to the Al(OH)PcOC solution, the intensity of B and Q bands slightly increased (Table 1). When albumin was added to the Al(OH)PcOC solution, the absorbance of the high intensity Q band went up by 1.22%. The effect of the albumin or amino acids on the absorbance of the Q band is as follows: albumin > glycine > l-serine > l-cysteine > l-tryptophan > l-histidine. The UV–vis spectra of Al(OH)PcOC presented in Fig. 3 showed that monomeric form of this complex predominates in a solution at pH 8.0. The ratio of the absorbances of the main Q band to the Soret band, and the ratio of absorbances of the main Q band to the vibronic band is constant, irrespective of which amino acid was added (ca. 2.4 for* A*_λmax_ = 691.5 nm/*A*_λmax_ = 360 nm and 5.8 for* A*_λmax_ = 691.5 nm/*A*_λmax_ = 621.5 nm). The reason for the small increase in band intensity may be the interaction with amino acids. This hypothesis was checked by TD-DFT calculations. Binding of Al(OH)PcOC to l-tryptophan and albumin was investigated by spectrofluorometry research (see Supplementary Materials). The value of* n* (number of binding sites) obtained suggests that the aluminum phthalocyanine derivative creates a 1:1.6 adduct with l-tryptophan.

**Table 1:** Absorbance values of aluminum octacarboxyphthalocyanine hydroxide [Al(OH)PcOC] and its complexes with albumin or amino acids. Concentrations of Al(OH)PcOC, amino acids and albumin were 5 × 10^−6^ , 2 × 10^−3^ mol/dm^3^, and 0.27 mg/ml, respectively

Compound	B band (at 360 nm)	Q band (at 691.5 nm)	Vibronic band (at 621.5 nm)
Al(OH)PcOC	0.519	1.229	0.210
Al(OH)PcOC + l-histidine	0.524	1.232	0.214
Al(OH)PcOC + l-tryptophan	0.526	1.233 (692 nm)	0.212 (622 nm)
Al(OH)PcOC + l-cysteine	0.525	1.234	0.214
Al(OH)PcOC + l-serine	0.523	1.238	0.214
Al(OH)PcOC + glycine	0.523	1.239	0.212
Al(OH)PcOC + albumin	0.529	1.244	0.215

### Photostability of Al(OH)PcOC and its complexes with amino acids

**Fig. 4:**
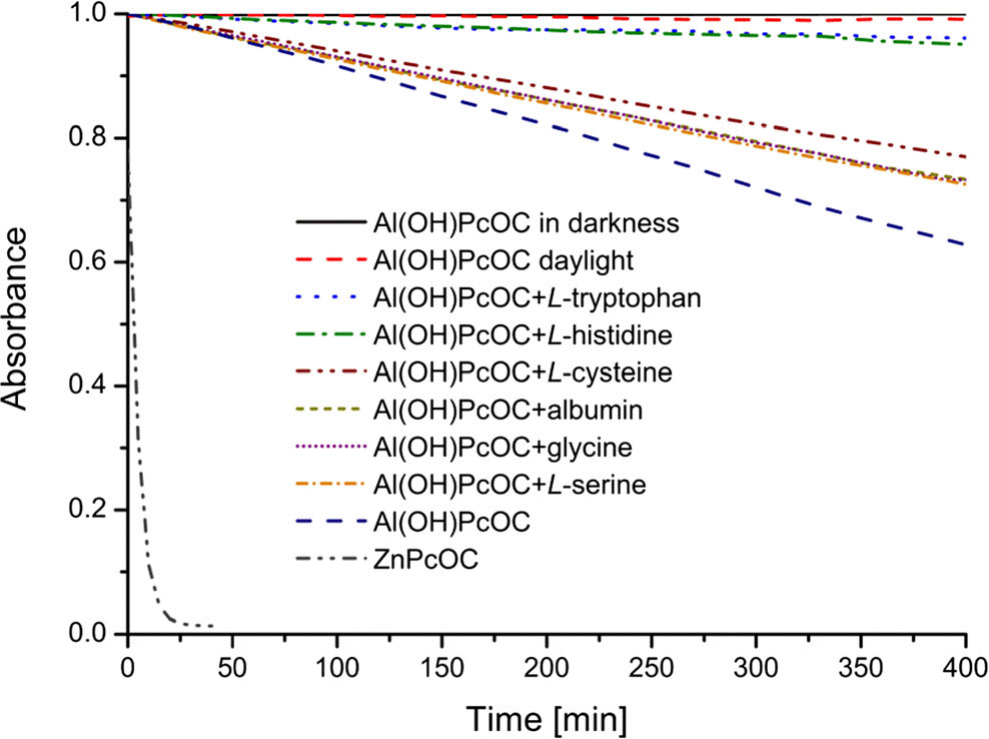
Kinetic curves of photolyzed Al(OH)PcOC and its complexes with albumin or with amino acids (glycine, l-cysteine, l-serine, l-histidine, l-tryptophan) in the phosphate buffer (pH 8.0) with exposure to red light-685 nm; ZnPcOC and Al(OH)PcOC concentration = 5 × 10^−6^ mol/dm^3^; albumin concentration = 0.27 mg/ml; concentration of amino acids =2 × 10^−3^ mol/dm^3^; measurements at λ_max_ = 691.5 nm for Al(OH)PcOC and λ_max_ = 688 nm for ZnPcOC

In the majority of applications, especially in PDT, the endurance of dyes to photobleaching plays a crucial role. Experimentally, photodegradation of Pcs is characterized by a decrease in the intensity of both the B and Q bands. The absorbance changes over time for the main Q band (λ_max_ = 691.5 nm) of the Al(OH)PcOC solution (pH 8.0) were examined upon exposure to daylight, visible light-685 nm, and for a solution “in darkness” (Fig. 4). Furthermore, photodegradation kinetic experiments were conducted for samples containing, beside Al(OH)PcOC, also albumin or amino acids. The results for the solutions irradiated with visible light are also shown in Fig. 4.

The kinetic curves indicate that Al(OH)PcOC is very stable when it was kept “in darkness”, while the intensity of the Q band decreases by degrees upon exposure of the samples to daylight and red light. However, when irradiated with red light, decrease of the absorbance is much faster (almost 40-fold faster compared to the solution exposed to daylight). Photodegradation by red light-685 nm can be characterized as a first-order reaction, the same as is the case of UV light [35]:

$$ A(t)={A}_0 \exp \left(-{k}_e\cdot t\right) $$

where *A* and *A*_0_ are absorbances of the Q band for octacarboxyphthalocyanines, respectively, for time *t* and *t* = 0, *k*_e_ (effective reaction rate constant). The received values of *k*_e_ comparing the photostability of the studied octacarboxyphthalocyanines are shown in Table 2. The photolysis of ZnPcOC complex occur by the mechanism of first-order reaction and the resulting *k*_e_ shown in [34]. Also, Schnurpfeil and co-workers presented the photo-oxidative stability of many complexes of zinc phthalocyanine; their *k*_*e*_ values were determined on the basis of the kinetics of the first-order reactions [36]. The rate constant of the irradiation of ZnPcOC complex under LED illumination was determined; its value was 0.1870 1/min. The value of the rate constant for this complex is much higher than was presented in [34] because a different dose of radiation was used. In the case of Al(OH)PcOC, complex dependence A = f(t) was linear (Fig. 4), indicating a 0-order reaction. For this complex, *k*_e_ was determined from the equation A_(t)_ = A_0_−kt (Table 2).

**Table 2:** Values of *k*_e_ (effective reaction rate constant) for Al(OH)PcOC and complexes of Al(OH)PcOC with amino acids or albumin. Concentrations of Al(OH)PcOC, amino acids and albumin were 5 × 10^−6^ , 2 × 10^−3^ mol/dm^3^, and 0.27 mg/ml, respectively

Compound	*k*_e_ × 10^9^ [$\frac{ m ol}{d{ m}^3\cdot \min }$]	
	Red light (685 nm)-irradiation	Daylight-irradiation
Al(OH)PcOC	6.06	0.16
Al(OH)PcOC + l-serine	4.93	0.11
Al(OH)PcOC + glycine	4.89	0.09
Al(OH)PcOC + l-cysteine	4.32	0.07
Al(OH)PcOC + l-histidine	0.84	0.07
Al(OH)PcOC + l-tryptophan	0.64	0.06
Al(OH)PcOC + albumin	4.34	0.07
	*k*_e_ × 10^2^ [min^−1^]	
ZnPcOC	18.70	0.35

**Fig. 5a,b:**
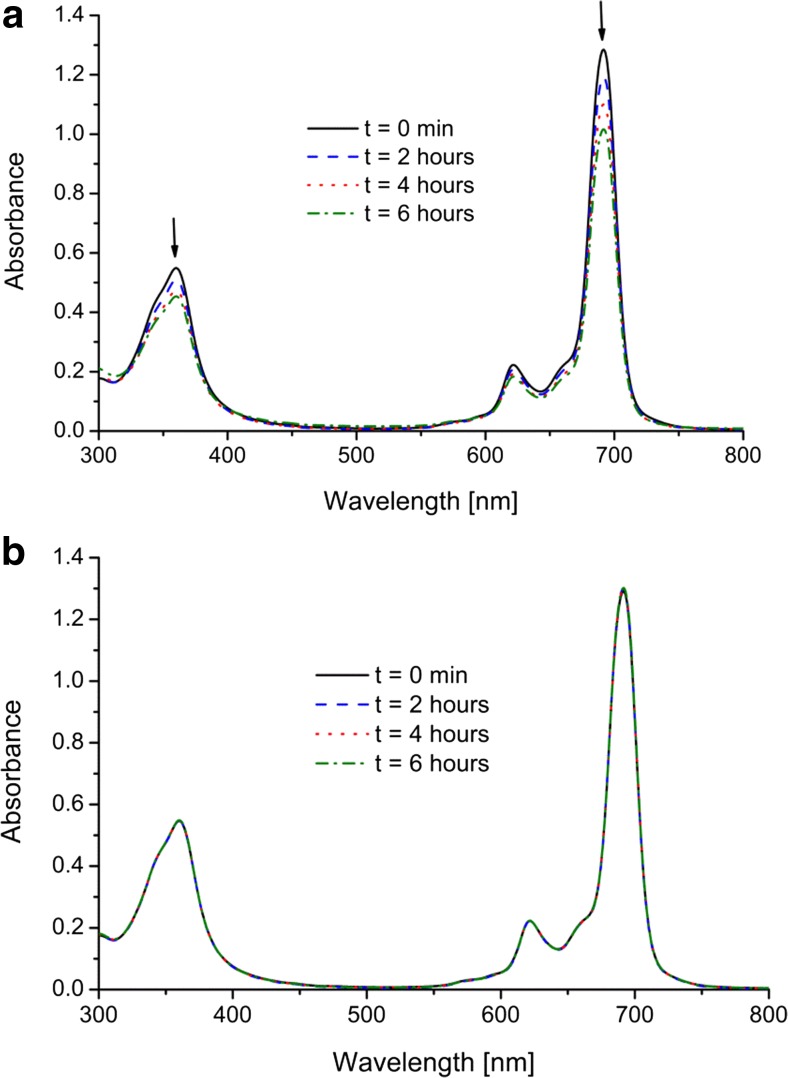
UV–vis spectra of Al(OH)PcOC solution (pH 8.0) after adding l-cysteine. Concentrations of Al(OH)PcOC, amino acids were 5 × 10^−6^ and 2 × 10^−3^ mol/dm^3^, respectively.** a** Visible light–685 nm,** b** daylight

From the results of this study, it can be seen that addition of amino acids to the solution increases the photostability of aluminum phthalocyanine hydroxide. When irradiated with red light (685 nm), amino acids cause a decrease in the *k*_*e*_ of Al(OH)PcOC photodegradation. However, the largest (up to ninefold) decrease in *k*_*e*_ was observed in the case of aromatic amino acids. Analogously, the presence of albumin in Al(OH)PcOC solution also causes a decrease of *k*_*e*_ of Al(OH)PcOC photodegradation. (Table 2). The slight increase in photostability of Al(OH)PcOC in the presence of albumin or amino acids was also observed for solutions irradiated with daylight. Figure 5 shows intensity changes in time for Al(OH)PcOC with l-cysteine for a solution exposed to red light–685 nm (Fig. 5a) and daylight (Fig. 5b).

Immediately after adding l-cysteine to the Al(OH)PcOC solution (t = 0 min), in the both cases: the solution exposed to daylight and red light–685 nm, two bands were observed in the Q-band region: a monomeric band at λ_max_ = 691.5 nm, and a vibronic band at λ_max_ = 621.5 nm, while the B band was observed at λ_max_ = 360 nm. During interaction studies of Al(OH)PcOC with albumin or amino acids, in both cases—the sample exposed to daylight and the sample exposed to red light–685 nm—the location of the B and Q bands does not change with time. However, red light (685 nm) influences their absorbance only slightly. For the Al(OH)PcOC solution with l-cysteine, which was irradiated with red light–685 nm, after 6 h, the absorbance of the Q band (λ_max_ = 691.5 nm) decreased by 20.9% and the Soret band by 17.4%. On the other hand, for the solution exposed to daylight, after 6 h, the intensities of the two bands did not change with time (the sample is not sensitive to daylight). The effect of amino acids on Al(OH)PcOC (at pH 8.0) placed "in darkness" was also studied. No change in band positions or intensities was observed.

Table 2 shows the rate constants of Al(OH)PcOC decomposition as result of exposure to red light (685 nm) and daylight. After irradiating the Al(OH)PcOC with 685 nm light, the effective photolysis rate constant is about 40 times higher than for the solution exposed to daylight (Table 2). The values of the effective reaction rate constants after adding albumin or amino acids to the Al(OH)PcOC solution were also determined. After adding an amino acid or albumin to the solution, in the case of samples exposed to red light (685 nm) the *k*_e_ constants changed in the following order: l-serine > glycine > albumin > l-cysteine > l-histidine > l-tryptophan. However, for the solution exposed to daylight, the *k*_e_ constant was lower, and changed in the same order as samples exposed to red light (Table 2, Fig. 4).

Our study showed that interaction of Al(OH)PcOC with albumin or selected amino acids decreases the photodegradation constant. This can be explained by the higher stabilization of the Al(OH)PcOC complex. The lowest value for *k*_e_ was noted for solutions containing Al(OH)PcOC and aromatic amino acids (l-histidine and l-tryptophan). The presence of the aromatic moiety in l-tryptophan (indole ring) and in l-histidine (imidazole ring) probably leads to a greater diversity of interactions between Al(OH)PcOC and aromatic amino acids.

### Calculated structures of Al(OH)PcOC–amino acid complexes

The experimental results presented in this study indicate that the photostability of Al(OH)PcOC phthalocyanine increases in the presence of amino acids. To explain this phenomenon, we performed full geometry optimization of phthalocyanine complexes with amino acids both in vacuum and in water using DFT methods. This type of complex may be axial or equatorial. Equatorial complexes are stabilized by H-bond formation between the Al(OH)PcOC carboxyl group and an –COOH or –NH_2_ group of the amino acid. Complexes of Al(OH)PcOC with axially coordinated amino acid in zwitterion form owe their stability to the interaction between the –COO^−^ group and the Al ion. These calculations allowed the effect of the amino acid on the structure of phthalocyanine to be determined, and the interaction energies with selected amino acids to be estimated. The possibility of interaction with side chains was also taken into account in the case of histidine. For both types of studied complex, the starting geometry was built up with a zwitterion form of amino acid. During geometry optimization of equatorial complexes, proton transfer from the amino group to the carboxyl group of the amino acid occurs.

**Fig. 6:**
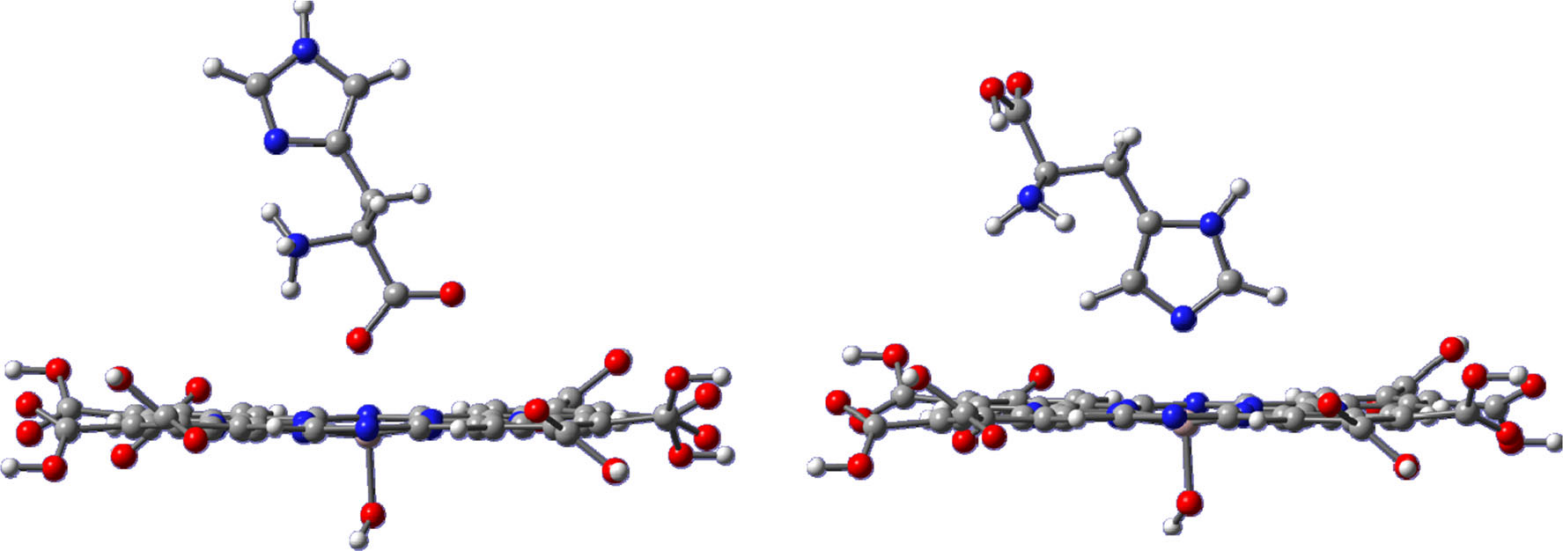
Two types of axial Al(OH)PcOC– l-histidine complexes. Structures obtained at B3LYP/6-31G(d) level

Figure 6 shows the B3LYP calculated structures of Al(OH)PcOC axial complexes with l-histidine, and Table 3 lists BSSE-corrected interaction energies and selected interatomic distances of optimized axial complexes structures.

**Table 3:** Energies of interactions and selected interatomic distances for axial Al(OH)PcOC:amino acid complexes obtained by B3LYP/6-31G(d) method

Complex	*E*_int _(kcal mol^−1^)	Al–O (OH) length [Ǻ]	Al ⋯O (N) distance [Ǻ]	Al–N length [Ǻ]	*d*(Al ⋯cOC plane) [Ǻ]
Vacuum					
Al(OH)PcOC	-	1.736	-	2.005	0.549
Al(OH)PcOC + glycine	15.2	1.770	2.175	1.989	0.265
Al(OH)PcOC + l-cysteine	16.3	1.771	2.151	1.989	0.255
Al(OH)PcOC + l-serine	18.6	1.774	2.122	1.989	0.241
Al(OH)PcOC + l-histidine	23.5	1.778	2.083	1.989	0.211
	10.5 (N)	1.770 (N)	2.336 (N)	1.988 (N)	0.245 (N)
Water					
Al(OH)PcOC	-	1.747	-	2.002	0.536
Al(OH)PcOC + glycine	23.9	1.806	2.024	1.987	0.152
Al(OH)PcOC + l-cysteine	24.6	1.801	2.020	1.987	0.152
Al(OH)PcOC + l-serine	24.6	1.807	2.018	1.987	0.147
Al(OH)PcOC + l-histidine	26.3	1.809	2.007	1.988	0.142
	14.7 (N)	1.800 (N)	2.202 (N)	1.987 (N)	0.160 (N)

The energy of interaction between Al(OH)PcOC and amino acids in axial complexes in vacuum were in the range 15–24 kcal mol^−1^. The Al ion and oxygen of the amino acid –COO^−^ group are separated by 2.18–2.08 Å, and this distance diminishes with increasing interaction energy. The shortest Al⋯O distance and the strongest interaction occurs for the adduct with l-histidine. Additionally, l-histidine can create another complex with Al(OH)PcOC via the nitrogen atom in the imidazole ring (Fig. 6). The parameters of this complex are also shown in Table 3, marked with N. This interaction is much weaker and its energy is about 11 kcal mol^−1^.

Formation of an Al(OH)PcOC–amino acid axial complex changes the structure of the phthalocyanine molecule. In the isolated molecule, the aluminum ion is “pulled out” of the plane of the molecule as a result of interaction with the –OH group. The distance of the aluminum atom from the plane, defined by the eight nitrogen atoms, is 0.549 Å. The interaction of the central metal with an amino acid –COO^−^ group results in pulling the Al(III) toward the center of the macrocycle. Consequently, the distance of Al from the plane of the phthalocyanine is clearly reduced. This distance decreases along with increasing of the interaction energy (*E*_int_). The shortest distance (0.211 Å) was observed for the complex with histidine. What is very interesting is that this structural effect is opposite to that found [34] for the ZnPcOC complexes with axially bound amino acids. In those latter complexes, the interaction with the amino acids produced a nonplanar structure of the macrocycle.

Solvent effects significantly change geometrical parameters and interaction energies in the axial complexes. The water environment reduces the Al–OH bond strength [in the isolated Al(OH)PcOC molecule, the Al…O(H) distance is 1.736 Å in the gas phase and 1.747 Å in water], and allows the creation of a stronger contact with the amino acid.* E*_int_ increases to 24–26 kcal mol^−1^. Also, in the complex with the histidine side chain, the interaction energy increased to 15 kcal mol^−1^. Moreover, the effect of water reduces the distance between an aluminum ion and the oxygen atom of amino acid–COO^−^ group (e.g., the Al⋯O distance is 2.175 Å and 2.024 Å in vacuum and in water, respectively, in the case of glycine). At the same time, a partial flattening of the phthalocyanine molecule occurs. For example, in complex with histidine, the distance between the Al ion and the plane defined by the eight nitrogen atoms decreases from 0.211 Å in vacuum to 0.142 Å in water.

**Fig. 7:**
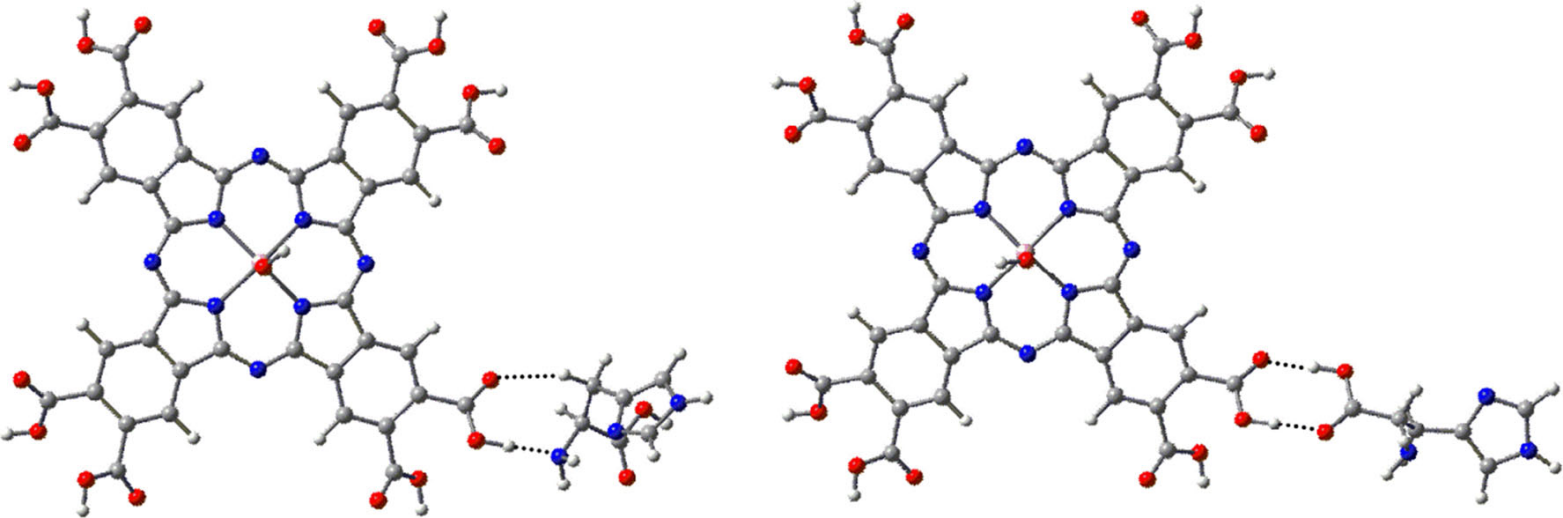
Structures of two types of equatorial complexes of Al(OH)PcOC with l-histidine calculated by B3LYP/6-31G(d) method.* Dotted lines* H-bonds

Other types of metal-ligand (ML) systems formed were aggregates formed via hydrogen bonds between phthalocyanine carboxyl groups and the –NH_2_ or COOH groups of amino acids (Fig. 7). Table 4 gathers the interaction energies and selected structural parameters of those complexes. It is apparent from our DFT results that formation of such equatorial complexes between phthalocyanine and amino acids does not change the properties of the macrocycle. It can therefore be concluded that the spectroscopic characteristics of such a complex should be almost the same as those of free phthalocyanines.

**Table 4:** Hydrogen bond energies (kcal mol^−1^) and their selected distances (Å) for equatorial Al(OH)PcOC–amino acid complexes

Complex	*E*_int_	OH⋯O	OH length	O⋯O	*d*(Al ⋯cOC plane) [Ǻ]
With two O-HEO bonds					
Vacuum					
Al(OH)PcOC + l-cysteine	18.7	1.680	1.005	2.685	0.548
Al(OH)PcOC + glycine	18.8	1.668	1.007	2.675	0.548
Al(OH)PcOC+ l-serine	19.3	1.680	1.005	2.685	0.548
Al(OH)PcOC+ l-histidine	18.8	1.659	1.008	2.667	0.548
Water					
Al(OH)PcOC + l-cysteine	19.4	1.674	1.006	2.679	0.538
Al(OH)PcOC + glycine	19.7	1.663	1.008	2.671	0.535
Al(OH)PcOC+ l-serine	19.5	1.672	1.007	2.678	0.536
Al(OH)PcOC+ l-histidine	19.7	1.659	1.008	2.667	0.535
With O-H···N and C^α^-H···O/O-H···N bonds					
	*E*_int_	OH⋯N	OH length	O⋯N	*d*(Al ⋯cOC plane) [Ǻ]
Vacuum					
Al(OH)PcOC + l-cysteine	13.4	1.725	1.018	2.741	0.548
Al(OH)PcOC + glycine	14.3	1.702	1.022	2.723	0.548
Al(OH)PcOC+ l-serine	15.6	1.689	1.026	2.715	0.548
Al(OH)PcOC+ l-histidine	18.4	1.636	1.040	2.675	0.548
	22.3^a^	1.749	1.011	2.711	0.548
Water					
Al(OH)PcOC + l-cysteine	14.6	1.647	1.036	2.682	0.536
Al(OH)PcOC + glycine	16.8	1.618	1.044	2.661	0.535
Al(OH)PcOC+ l-serine	17.2	1.611	1.047	2.656	0.535
Al(OH)PcOC+ l-histidine	19.5	1.569	1.061	2.628	0.535
	30.2^a^	1.652	1.033	2.664	0.535

^a^The equatorial complex is stabilized due to the presence of two O-H···N hydrogen bonds formed by the NH_2_ group and with a nitrogen atom in the l-histidine side chain

The energy of two O–H⋯O H-bonds between the –COOH group of the amino acid and Al(OH)PcOC in equatorial complexes is about 19 kcal mol^−1^ for all studied amino acids. The effect of water on the structures and interaction energies of those equatorial complexes is much smaller than in the case of axial complexes. Interaction energy in an aqueous medium increases slightly (by less than 1 kcal mol^−1^).

Somewhat weaker hydrogen bonds are formed between the acid group of the phthalocyanine and the amino group of the amino acid. Their energy is the range of 13–18 kcal mol^−1^ in the gas phase, and 15–19 kcal mol^−1^ in a water environment. On the other hand, the interaction with the nitrogen atom in the histidine side chain is distinctly stronger (22.3 kcal mol^−1^ and 30.2 kcal mol^−1^ in gas the phase and in water, respectively), which is in agreement with experimental findings that histidine enhances the photostability of the studied phthalocyanine more effectively than cysteine, glycine or serine.

The DFT-predicted interaction energies between Al(OH)PcOC and amino acid molecules in both types of complexes in the gas phase are similar. Interestingly, in aqueous environment, axial complexes are favored by about 5 kcal mol^−1^ over equatorial complexes. However, it should be noted that it not only the energy factor that affects the formation of the Al(OH)PcOC complexes with amino acids, the entropy factor is probably also important. It seems that steric requirements are much more stringent in the case of axial complexes (especially considering that the aluminum ion is pulled out from the phthalocyanine plane by the –OH group). On the other hand, peripheral carboxylic groups are more easily accessible, and can interact with both the acid and amino groups of the amino acids. Beside, Al(OH)PcOC can bind axially only one amino acid molecule. In contrary, multiple binding is possible for equatorial complexes.

**Fig. 8:**
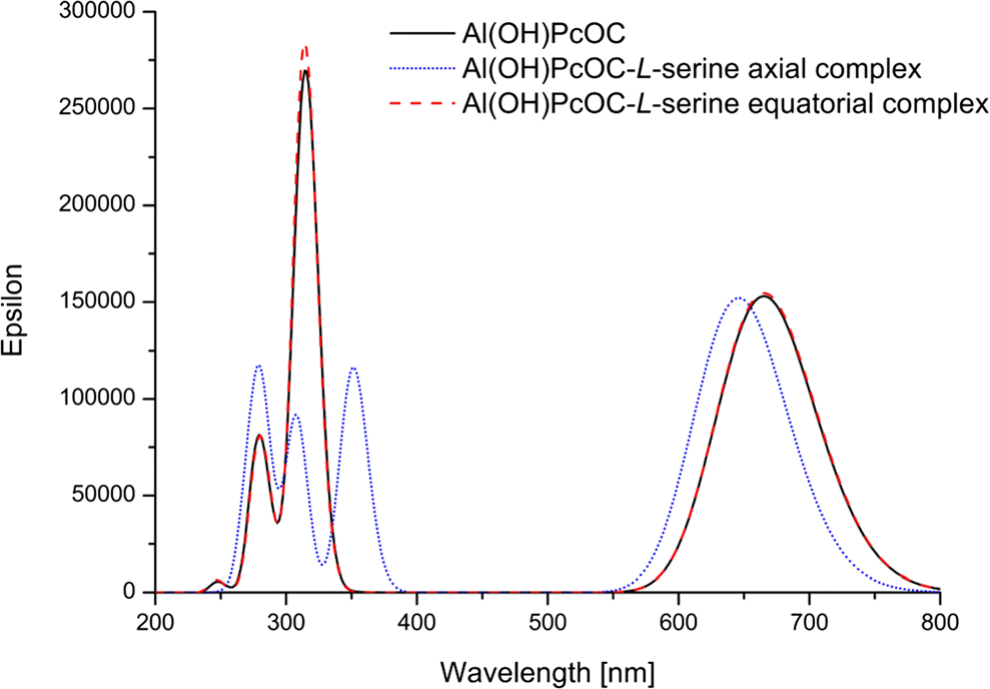
UV–Vis spectra of Al(OH)PcOC and its complexes with l-serine in water calculated by the TD-DFT/CAM-B3LYP/6-31G(d) method

To determine the effect of interaction with amino acids on phthalocyanine optical properties, the UV–vis spectra for Al(OH)PcOC and its two complexes with l-serine were calculated (Fig. 8) at TD-DFT/CAM-B3LYP/6-31G(d) level in the water environment modelled by the PCM method. According to our TDDFT results, there are two intense bands at 668 nm and 314 nm for Al(OH)PcOC. The band positions closely reproduce the experimental spectra. The spectra of Al(OH)PcOC and its equatorial complex with l-serine are almost identical. The interaction with amino acids in such a complex results in a slightly increased intensity of both bands. This phenomenon is also observed experimentally in the electronic spectrum of Al(OH)PcOC in the presence of amino acids. In the case of the axial complex, splitting of the Soret band is predicted theoretically (Fig. 8), but not observed experimentally.

## Conclusions

In summary, we investigated the impact of albumin and several coded amino acids on the electronic absorption spectra and photostability of Al(OH)PcOC by means of UV–vis spectroscopy and DFT calculations. Our investigation reveals that, in solutions containing an amino acid or albumin, no change in the positions of the bands in the UV–vis spectrum was observed for the studied compound, Al(OH)PcOC. Instead, a slight increase in the absorbance of the bands in the Q and B regions occur. As a result of exposure to red light, Al(OH)PcOC decomposes, as revealed by a decrease in band intensity in both regions. The photostability of the phthalocyanine is higher upon the addition of amino acids or bovine serum albumin to the solution. An increased photostability in the presence of amino acids and albumin was also observed for zinc octacarboxyphthalocyanine [34]. It is concluded that the studied amino acids do not degrade zinc and aluminum phthalocyanines. Therefore, the investigated compounds could be used as potential photosensitizers. DFT/ B3LYP/6-31G(d) // TD-DFT/CAM-B3LYP/6-31G(d) calculated UV–vis spectra for equatorial complex of Al(OH)PcOC with amino acids closely resembled experimental spectra. This indicates the absence of the axial complex in solution. The structures of equatorial Al(OH)PcOC complexes with selected amino acids are stabilized by hydrogen bonds formed between carboxylic groups of phthalocyanine and amino acid. The energy of such interactions is equal to 19 kcal mol^−1^ and increases slightly in polar environments.

## Electronic supplementary material

Below is the link to the electronic supplementary material.

ESM 1 (DOC 902 kb)
