# Supplementary material for: Interactions of amino acids with aluminum octacarboxyphthalocyanine hydroxide. Experimental and DFT studies
Source: J Mol Model. 2017 Feb 4;23(2):51. doi: 10.1007/s00894-017-3222-2 (PMC5306055; doi:10.1007/s00894-017-3222-2)
Supplement: Supplementary file 1 — (DOC 902 kb) [file 894_2017_3222_MOESM1_ESM.doc]

**Interactions of amino acids with aluminum octacarboxyphthalocyanine hydroxide. Experimental and DFT studies.**

Marta Kliber-Jasik^1*^, Małgorzata A. Broda^1^, Anna Maroń^2^, Joanna Nackiewicz^1^

*^1^Department of Physical Chemistry and Molecular Modeling, Faculty of Chemistry, University of Opole, Oleska 48, Opole 45-052, Poland*

*^2^Department of Crystallography, Institute of Chemistry University of Silesia 9th Szkolna St., Katowice 40-006, Poland*

^*^Corresponding author: e-mail address: mkliber@uni.opole.pl.

**SUPPLEMENTARY MATERIALS**

**Experimental**

***Materials***

The phosphate buffer (pH 8.0) was prepared using grade sodium hydrogen phosphate dodecahydrate (*Na*_2_*HPO_4_*·12*H*_2_*O*) and sodium dihydrogen phosphate dihydrate, (*NaH*_2_*PO_4_*·2*H*_2_*O*), POCH Gliwice. Bovine serum albumin (BSA) – crystallized, freeze-dried, min. albumin content of 98%, was purchased from POCH Gliwice; glycine from Argon – Łódź; *L*-cysteine from Loba Chemie; *L*-histidine from Chemapol, *L*-serine from Reanal; *L*-tryptophan from Reanal. Pyromellitic dianhydride (benzene-1,2,4,5-tetracarboxylic dianhydride), DBU, Al_2_O_3_ a.p. Merck, urea, AlCl_3_, acetone, HCl, H_2_SO_4_, NaOH a.p. grade were purchased from POCH Gliwice. The Britton–Robinson buffer was prepared from acetic acid, orthophosphoric acid, boric acid and sodium hydroxide, all a.p., POCh Gliwice.

***Measurements***

Ultraviolet–Visible (UV–Vis) spectra were recorded using Jasco V-650 spectrophotometer. The spectra were measured at 25°C or 37°C in 10 mm quartz cells. The exposition of the solutions on visible light was carried out using the LED lamp by OPTEL_OPOLE_, λ_max_=685nm, intensity 6.4 mW/cm^2^ at 25°C or 37°C. For comparison, the solutions were also exposure to daylight (UV portion 2µW/cm^2^). All measurements were conducted in the phosphate buffer at a pH 8.0. Julabo F25 thermostat was used for temperature control. A CP-315_M_ pH-meter from Elmetron Poland was used for pH measurements. The research was carried out for Al(OH)PcOC concentration 5·10^-6^mol/dm^3^ and 1·10^-5^ mol/dm^3^. In the sample amino acid concentration was 2·10^-3^mol/dm^3^ and albumin concentration was 0.27mg/ml. Albumin was dissolved in 0.9% NaCl solution. Steady-state luminescence spectra of solution samples were measured on the FLS-980 fluorescence spectrophotometer equipped with a 450 W Xe lamp and high-gain photomultiplier PMT + 500nm (Hamamatsu, R928P) detector. The emission spectra were measured in 10.00 mm quartz cells.  Fluorescence excitation spectra were recorded on Hitachi F-7000 Spectrofluorometer.

***Theoretical calculations***

Unconstrained geometry optimization of the molecular structures of Al(OH)PcOC complexes with selected amino acids (in the zwitterionic form) were performed using the Gaussian 09 program [26]. Density functional theory (DFT) method was applied using the hybrid functional B3LYP [27,28] and the 6-31G(d) basis set on all the atoms. This widely used and efficient B3LYP functional demonstrated high accuracy in many real-world problems [29,30] and has been recognized as probably the best compromise between computational cost and accuracy for relatively large systems, such as metalloporphyrins or metallophthalocyanines. This was also demonstrated in our recently published works [31,32]. To study the solvent effects on selected properties of Al(OH)PcOC complexes the polarized continuum model (PCM) [33,34] within the self-consistent reaction field (SCRF) was used to model the water phase. In all cases, fully optimized structures are characterized by computing second energy derivatives. The energy of interaction was evaluated from energy differences between complex and its individual components and was corrected using the counterpoise (CP) procedure of Boys and Bernardi [35]. The energies and intensities of electronic transitions were calculated employing TD-DFT methods at the CAM-B3LYP/6-31G(d) [36] level of theory. The spectra were calculated in water for 40 excited states by employing the polarizable continuum model (PCM method).

**Binding of aluminium octacarboxyphthalocyanine hydroxide complex with amino acid and albumin**

Research of the fluorescence quenching of the amino acid (tryptophan) and albumin was conducted by the addition of increasing concentrations of aluminum octacarboxyphthalocyanine hydroxide (Al(OH)PcOC) at a fixed concentration of amino acid and albumin. The concentration of Al(OH)PcOC varied in the range of from 1.62⋅10^-6^ to 8.08⋅10^-6^ M, while that concentration of tryptophan was kept constant at 2⋅10^-4^ M. However, in the case of measuring the albumin, concentration of Al(OH)PcOC changed in the range of from 2.80⋅10^-6^ to 1.00⋅10^-5^ M, while that concentration of albumin was kept constant at 2 mg/ml. The measurements were carried out in buffers (phosphate buffer) at constant pH = 8.00. A sample of tryptophan was excited at 290 nm, and the fluorescence spectra were measured in the range 295 to 500 nm. The fluorescence of tryptophan exhibits at λ_max_=347 nm (Fig. 1). Fig. 1b shows the change in the fluorescence emission spectra of tryptophan after the addition of different concentrations of aluminum octacarboxyphthalocyanine hydroxide. Tryptophan and Al(OH)PcOC complex exhibits common fluorescence quenching (Fig. 1). Decreasing fluorescence intensity of tryptophan with increasing concentration of Al(OH)PcOC complex was used to determine the binding constants (K_b_) and the number of binding sites (n) on tryptophan, according to Eq. 1 [23]

$\log\left[\frac{(F_{0}-F)}{(F-F_{\infty}}\right]=\log K_{b}+n\log[Pc]$ (1)

where F_0_ and F are the fluorescence intensities of tryptophan in the absence and presence of Al(OH)PcOC complex; F_∞_, the fluorescence intensity of tryptophan saturated with Al(OH)PcOC complex; K_b_, the binding constant; ‘n’, the number of binding sites on a tryptophan molecule; and [Pc] the concentration of Al(OH)PcOC complex. Plots of log[(F_0_-F)-(F-F_∞_)] against log [Pc]` provide the values of ‘n’ and K_b_. A linear relationship was obtained by the equation y=1,6101+8,5228 (R^2^=0,9969). The value of K_b_ is 3.34⋅10^8^ M^-1^ and it is higher than the K_b_ for interaction BSA-MPC, and the value of ‘n’ is 1.61 [48]. This suggests that the resulting complex Al(OH)PcOC strongly associated with tryptophan, further Al(OH)PcOC forms a tryptophan adduct 1: 1.6. The BSA exhibits the fluorescence at 334 nm and it can be attributed to tryptophan residues in the molecule. (Fig. 1b). The number of binding sites indicates the Al(OH)PcOC and BSA create adducts with 1:1.6 stoichiometry. The value of K_b_ is 1.06⋅10^8^ M^-1^.


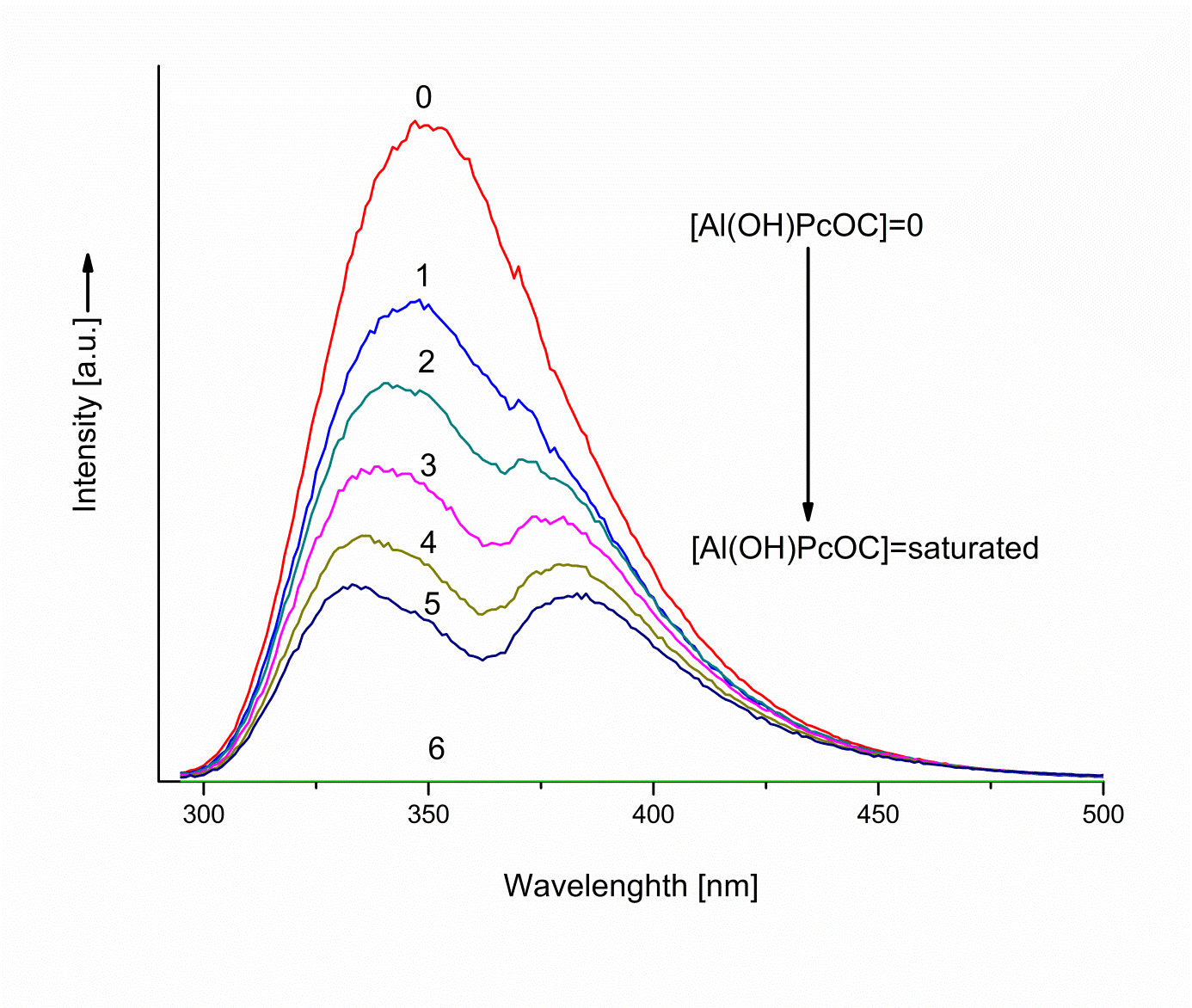


Fig. 1a Fluorescence emission spectral changes of tryptophan (c=2⋅10^-4^ M) on addition of varying concentrations of Al(OH)PcOC complex in phosphate buffered solution (pH=8,0). [Al(OH)PcOC]: 1) 2,0⋅10^-6^ M; 2) 4,0⋅10^-6^M 3) 6.0⋅10^-6^ M 4) 8.0⋅10^-6^M; 5) 1,0⋅10^-5^M; 6) saturated with Al(OH)PcOC


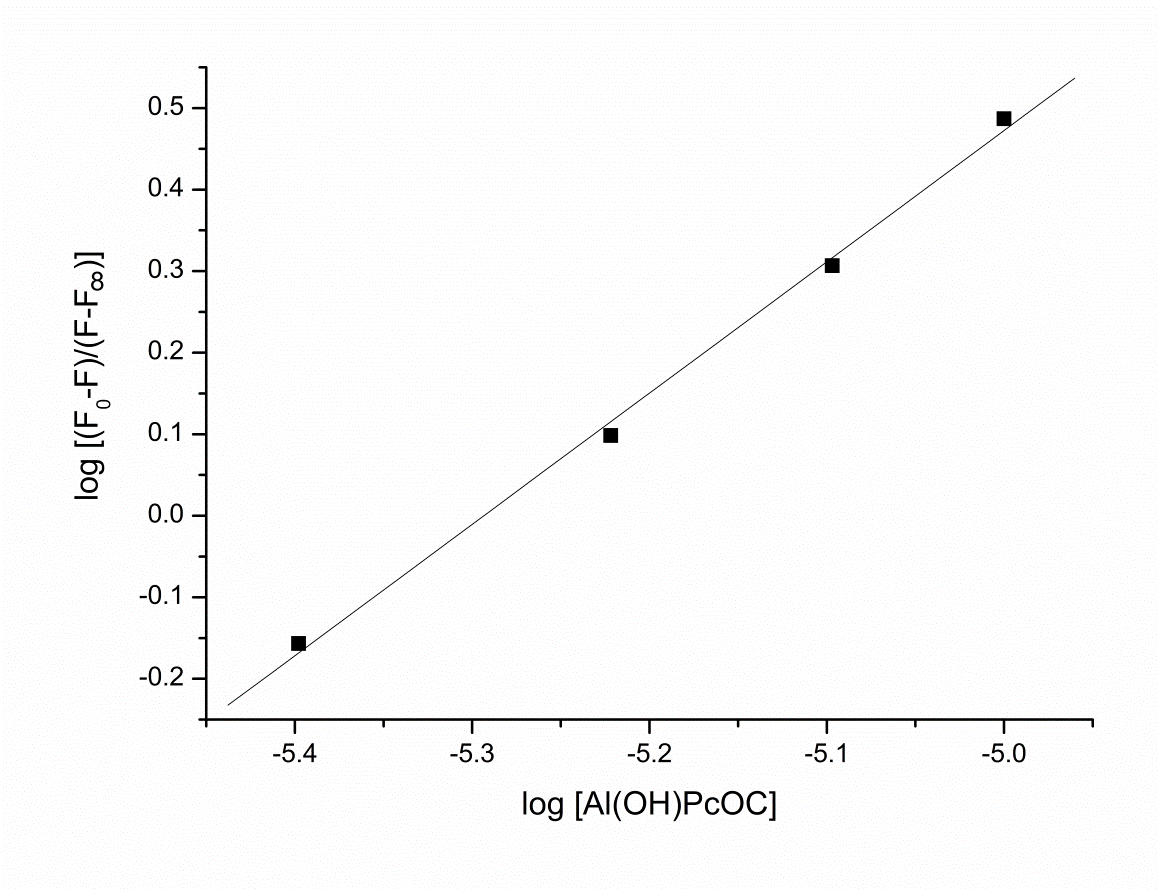


Fig. 2a Determination of Al(OH)PcOC- tryptophan binding constant. [tryp] c=2⋅10^-4^ M; [Al(OH)PcOC]: 2,0⋅10^-6^ M; 2) 4,0⋅10^-6^M 3) 6.0⋅10^-6^ M 4) 8.0⋅10^-6^M; 5) 1,0⋅10^-5^M; in phosphate buffered solution (pH=8,0)


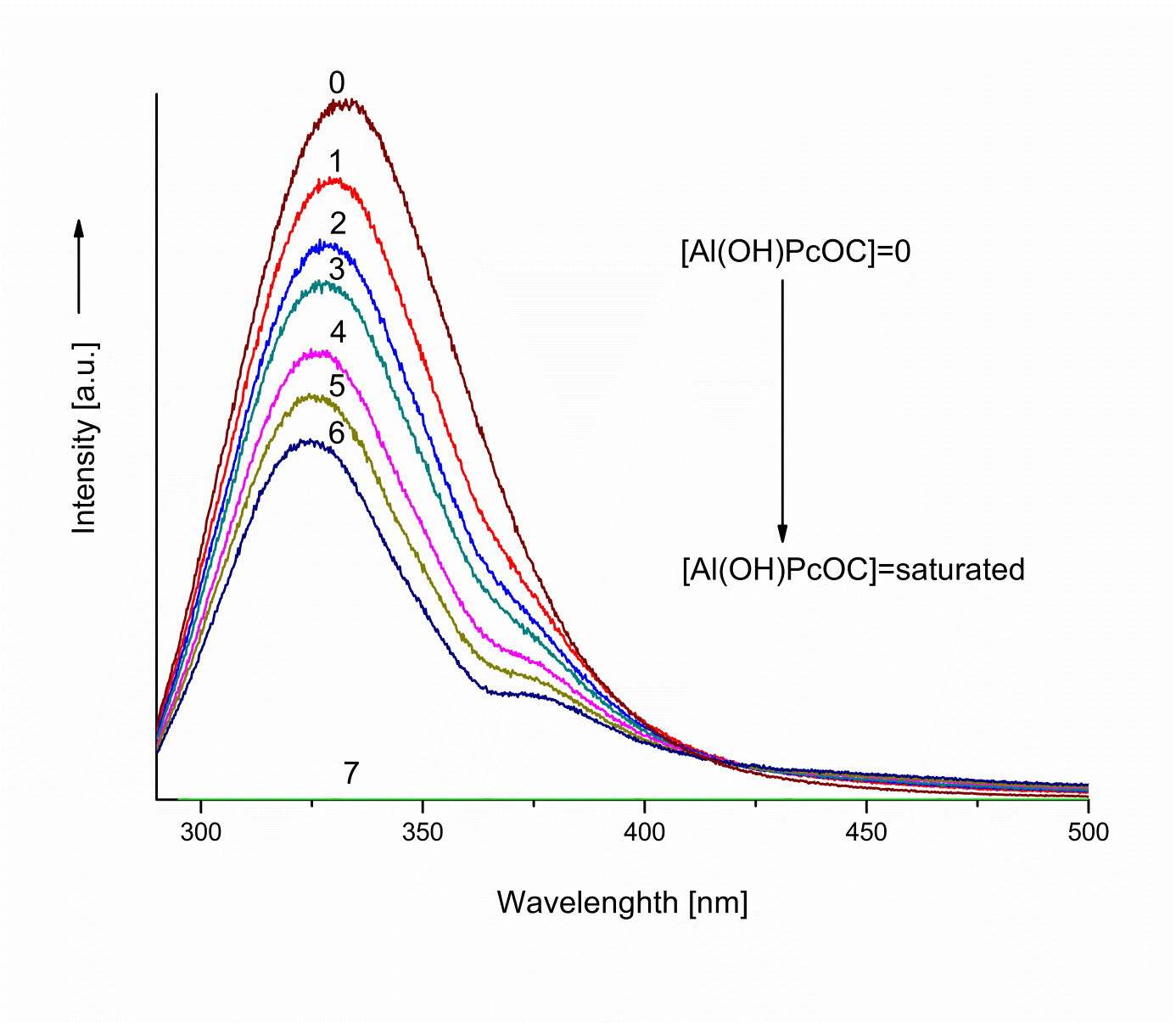


Fig. 1b Fluorescence emission spectral changes of albumin (c=2 mg/ml) on addition of varying concentrations of Al(OH)PcOC complex in phosphate buffered solution (pH=8,0). [Al(OH)PcOC]: 1) 2,8⋅10^-6^ M; 2) 4,0⋅10^-6^M 3) 4,8⋅10^-6^ M 4) 6,8⋅10^-6^M; 5) 8,0⋅10^-6^M; 6) 1,0⋅10^-5^M; 7) saturated with Al(OH)PcOC


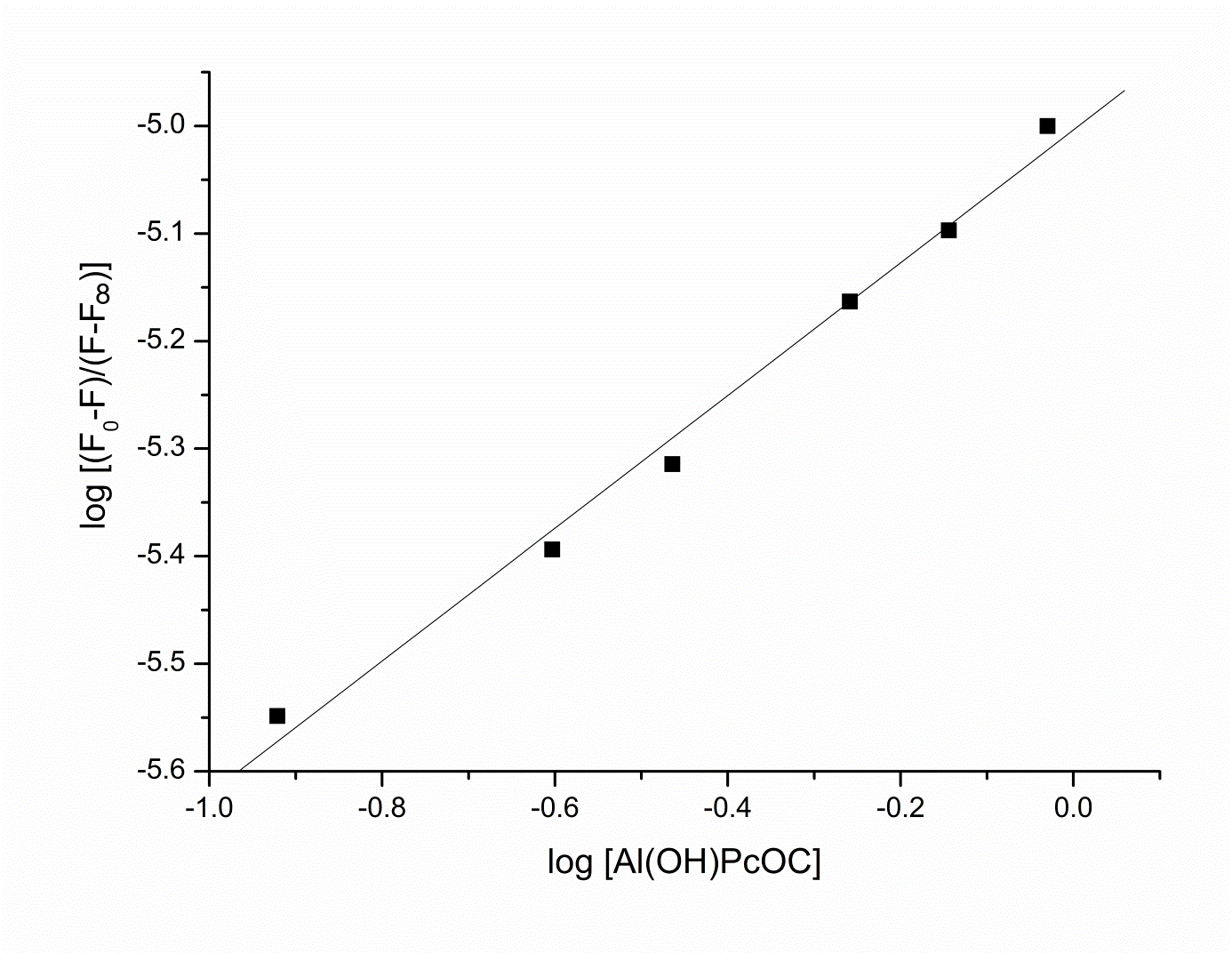


Fig. 2b Determination of Al(OH)PcOC- albumin binding constant. [albumin] c=2 mg/ml; [Al(OH)PcOC]: 1) 2,8⋅10^-6^ M; 2) 4,0⋅10^-6^M 3) 4,8⋅10^-6^ M 4) 6,8⋅10^-6^M; 5) 8,0⋅10^-6^M; 6) 1,0⋅10^-5^M; in phosphate buffered solution (pH=8,0)

**REFERENCES**

1. Idowu M, Nyokong T (2008) Photosensitizing properties of octacarboxy metallophthalocyanines in aqueous medium and their interaction with bovine serum albumin. J Photochem Photobiol A 200:396–401. doi: [10.1016/j.jphotochem.2008.09.003](http://dx.doi.org/10.1016/j.jphotochem.2008.09.003).
2. Gaussian 09, Revision B.01, Frisch MJ, Trucks GW, Schlegel HB, Scuseria GE, Robb
   MA, Cheeseman JR, Scalmani G, Barone V, Mennucci B, Petersson GA, Nakatsuji H, Caricato M, Li X, Hratchian HP, Izmaylov AF, Bloino J, Zheng G, Sonnenberg JL, Hada M, Ehara M, Toyota K, Fukuda R, Hasegawa J,. Ishida M, Nakajima T, Honda Y, Kitao O, Nakai H, Vreven T, Montgomery JA, Jr, Peralta JE, Ogliaro F, Bearpark M, Heyd JJ, Brothers E, Kudin KN, Staroverov VN, Kobayashi R, Normand J, Raghavachari K, Rendell A, Burant JC, Iyengar SS, Tomasi J, Cossi M, Rega N, Millam NJ, Klene M, Knox JE, Cross JB, Bakken V, Adamo C, Jaramillo J, Gomperts R, Stratmann RE, Yazyev O, Austin AJ, Cammi R, Pomelli C, Ochterski JW, Martin RL, Morokuma K, Zakrzewski VG, Voth GA, Salvador P, Dannenberg JJ, Dapprich S, Daniels AD, Farkas O, Foresman JB, Ortiz JV, Cioslowski J, Fox DJ. Gaussian, Inc., Wallingford CT 2009.
3. Becke AD (1993) Density‐functional thermochemistry. III. The role of exact exchange. J Chem Phys 98:5648–5652. doi: [10.1063/1.464913](http://dx.doi.org/10.1063/1.464913)
4. Lee C, Yang W, Parr RG (1988) Development of the Colle-Salvetti correlation-energy formula
    into a functional of the electron density. Phys Rev B 37:785–789. doi: 10.1103/PhysRevB.37.785
5. Singleton DA, Wang ZH (2005) Isotope Effects and the Nature of Enantioselectivity in the Shi Epoxidation. The Importance of Asynchronicity. J Am Chem Soc 127:6679–6685. doi: 10.1021/ja0435788
6. Schneebeli ST, Hall ML, Breslow R, Friesner R (2009) Quantitative DFT Modeling of the Enantiomeric Excess for Dioxirane-Catalyzed Epoxidations. J Am Chem Soc 131:3965–3973. doi: 10.1021/ja806951r
7. Man D, Słota R, Broda MA, Mele G, Li J (2011) Metalloporphyrin intercalation in liposome membranes: ESR study. J Biol Inorg Chem 16:173–181. doi: 10.1007/s00775-010-0715-1
8. Wang C, Yang G, Li J, Mele G, Słota R, Broda MA, Duan M, Vasapollo G, Zhang X, Zhang FX, (2009) Novel *meso*-substituted porphyrins: Synthesis, characterization and photocatalytic activity of their TiO_2_-based composites. Dyes Pigm 80:321–328. doi: [10.1016/j.dyepig.2008.08.008](http://dx.doi.org/10.1016/j.dyepig.2008.08.008)
9. Miertus S, Tomasi J (1982) Approximate evaluations of the electrostatic free energy and internal energy changes in solution processes. Chem Phys 65:239–245. doi: [10.1016/0301-0104(82)85072-6](http://dx.doi.org/10.1016/0301-0104(82)85072-6)
10. Tomasi J, Mennucci B, Cammi R (2005) Quantum Mechanical Continuum Solvation Models. Chem Rev 105:2999–3093. doi: 10.1021/cr9904009
11. Boys SF, Bernardi F (1970) The calculation of small molecular interactions by the differences of separate total energies. Some procedures with reduced errors. Mol Phys 19:553–566. doi: [10.1080/00268970110088901](http://dx.doi.org/10.1080/00268970110088901)
12. Yanai T, Tew DP, Handy NC (2004) A new hybrid exchange–correlation functional using the Coulomb-attenuating method (CAM-B3LYP). Chem Phys Lett 393:51–57. doi: [10.1016/j.cplett.2004.06.011](http://dx.doi.org/10.1016/j.cplett.2004.06.011)
13. Çamura M, Ahsena V, Durmuş M (2011) The first comparison of photophysical and photochemical properties of non-ionic, ionic and zwitterionic gallium (III) and indium (III) phthalocyanines. J. Photochem. Photobiol. A: Chem 219: 217–227. doi: [10.1016/j.jphotochem.2011.02.014](http://dx.doi.org/10.1016/j.jphotochem.2011.02.014)
